# Salacine as a Substitute for Quinine

**Published:** 1845-09

**Authors:** 


### SALACINE AS A SUBSTITUTE FOR QUININE.

Some experiments with *salacine* in intermittent fever, made at New Orleans, do not appear to have resulted very favorably, but others are being made in the Army on a more extensive scale, of which we hope to hear a more encouraging report. It is highly desirable that some substitute for the present expensive remedy in intermittents should be found, and especially since there is a probability that Great Britain may monopolize the trade in Peruvian bark, in which event the price of quinine will be still further advanced. The willow may be cultivated in this country to any extent, and the cost of salacine made very trifling, if it should prove to be an efficacious remedy. Y.

